# Inducing apoptosis of human colon cancer cells by an IGF-I D domain analogue peptide

**DOI:** 10.1186/1476-4598-7-17

**Published:** 2008-02-08

**Authors:** Shi Yu Yang, Kevin M Sales, Barry J Fuller, Alexander M Seifalian, Marc C Winslet

**Affiliations:** 1 University Department of Surgery, Royal Free & University College Medical School, University College London, Rowland Hill Street, London NW3 2PF, UK; 2 Royal Free Hampstead NHS Trust Hospital, London, UK; 3 University College Hospital, London, UK

## Abstract

**Background:**

The resistance of tumour cells to apoptosis is a major contributor to the limited effectiveness of chemotherapies. Insulin-like growth factor I (IGF-I) has potential to protect cancer cells from variety of apoptotic challenges. This study was carried out to investigate the effect of a novel IGF-I receptor antagonist on apoptosis in colon cancer cells.

**Results:**

We have designed and synthesised a novel antagonist of IGF-I receptor. The effect of this antagonist on human colon cancer cell proliferation was examined by a non-radioactive assay; the apoptosis was revealed by determining the activities of cellular caspases3/7, 8 and 9. The apoptosis pathways were investigated by examining the levels of pro-apoptosis proteins with Western blotting. Following 40 hours treatment with the novel antagonist peptide, colon cancer cell Caspase 3/7 activities increased 2–7 times; Caspase 8 activities increased 2–5 times and Caspase 9 increased 1.2–1.6 times. The proliferation of cancer cell was inhibited by 14–15%. The data showed that the antagonist induced colon cancer cell apoptosis and inhibited cancer cell proliferation. The different changes of Caspase 3/7, 8 and 9 activities suggested that the extrinsic pathways may play a major role in the antagonist peptide-induced apoptosis.

**Conclusion:**

This is the first report on this novel antagonist to induce human colon cancer cell apoptosis and inhibit cancer cell proliferation. These results suggest that IGF-I receptor antagonists may have the potential to be developed as a novel therapy for colon cancers in the future.

## Background

Worldwide, colorectal cancer accounts for almost one million new cases and causes a half million deaths annually [1]. In Europe colorectal cancer ranks second in frequency of new cases in both men and women and is the second leading killer after lung cancer [2]. Colorectal cancer is presently treated by surgical ablation, but many colorectal cancers are detected at a late stage when surgery cannot cure the disease. At least 40% of patients with colorectal cancer develop metastases; chemotherapy alone or in combination with radiotherapy can be used as an adjuvant therapy to surgery for more advanced disease [3]. However, these approaches are not highly effective against disseminated colorectal cancer [4]. New therapeutic strategies are needed for treatment of advanced or metastatic colorectal cancer.

The resistance of tumour cells to apoptosis is of major concern in cancer therapy. It is a major contributor to the limited effectiveness of current chemotherapeutic drugs. Several growth factors have been identified as regulators of cancer cell survival, and of these factors, insulin-like growth factor I (IGF-I) has been reported to have a potential to protect a broad range of cells from a variety of apoptosis challenges. IGF-I receptors are present on primary cell masses of human colon carcinomas and on colorectal cancer cell lines [5]. Colorectal carcinomas have a 10 to 50-fold increase in the level of IGF-I and IGF-II when compared to adjacent uninvolved colonic mucosa [6-8]. IGF-I stimulate growth of HT-29, LS411N LS513, SW480 and WiDr human colorectal carcinoma cell lines [9]. Accumulated data from laboratory experiments demonstrate that IGF-I and IGF-II are able to stimulate the growth of wide variety of cancer cells and to suppress apoptosis. Therefore the IGF system has become an attractive molecular target for anticancer therapies. Inhibition of the IGF-IR pathway, however, had not been successfully exploited as a major anticancer therapeutic strategy due to the lack of clinically applicable inhibitors of IGF-IR. Although some positive results have been obtained in recent *in vivo *studies using anti-IGF-IR antibodies to treat prostate cancer [10], the adverse effects of this therapy cannot be ruled out as it interferes with the systemic IGF system.

IGF-I is a 70 amino acid peptide which includes A, B, C and D domains. Functionally IGF-I has metabolic and mitogenic actions (which include anti-apoptosis and cellular survival functions). It has been shown that IGF-I regulates cellular proliferation, differentiation [11] and apoptosis [12] of intestinal epithelium cells. IGF-I fully protected HT-29-D4 colon carcinoma cells form apoptosis induced by tumour necrosis factors-α [12]. Using hybrid molecular and chemical modifications of constituent amino acid, it has been found that D domain and a tyrosine residue (Tyr-60) in the A domain play a decisive role for IGF-I binding to its receptor [13-15]. In this study we have designed and synthesised a novel antagonist of IGF type I receptor, which is an analogue of the IGF-I D domain (M1557 peptide). The following is a report regarding the effect of this IGF-I D domain analogue peptide on colon cancer cell apoptosis and proliferation.

## Methods

### IGF-I D domain analogue peptide (M1557) design and synthesis

**Figure 1:**
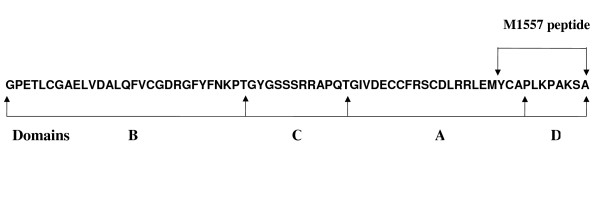
Human insulin-like growth factor I (IGF-I) sequences and the relation of M1557 peptide to insulin-like growth factor I domains.

The design and sequences of IGF-I D domain analogue peptide (M1557) is shown in Figure 1. Sequences of the peptide covered the entire IGF-I D domain plus 3 extra amino acid residues from the A domain. It was reported that the residual Try-60 in the A domain has a critical role for IGF-I binding to its receptor [15], therefore we included 3 extra amino acid residues (including Try-60) in the N-terminal of the peptide. The peptide was synthesised by Alta Bioscience (University of Birmingham) using solid phase peptide synthesis in the sequence Y (Acm) CAPLKPAKSA-amide. In order to avoid enzyme recognition and subsequent cleavage, the amino acids used were all D-isomer except Cysteine (Acm). Cysteine was protected with ACM intact attached. The peptide was purified by high-pressure liquid chromatography (HPLC). The purity and quality of the peptide were determined and controlled by mass spectrometer and HPLC.

### Reagents

Recombinant human IGF-I was purchased from Peprotech EC Ltd (UK). Caspase3/7, Caspase 8 and Caspase 9 activity assay kits and CellTiter-Glo luminescent kits were purchased from Promega (Madison, USA). Modified Lowry protein assay kit was purchased from Pierce Biotechnology (USA). Bax polyclonal antibodies were purchased from Santa-Cruz (USA). All cell cultural mediums, serums and antibiotics were purchased from GIBCO (UK).

### Cell lines and cultural conditions

The human colon cancer cell line (HT29) was purchased from European Collection of Cell Cultures (ECACC) and maintained with Growth Medium (GM, McCoy's 5A medium containing 2 mM glutamine, 10% foetal bovine serum and 1% penicillin and streptomycin) at 37°C with 5% CO_2_/95% air. Cells were used at passages 3–8 after their receipt from the supplier.

### Cancer cell apoptosis assay

Human colon cancer cells (HT-29) were seeded on white-walled 96 well plates with McCoy's 5A medium containing 2 mM glutamine, 10% foetal bovine serum and 1% penicillin and streptomycin at the density of 2 × 10^4 ^cells/well. The different concentrations (0, 0.001, 0.01, 0.1, 1 and 2 μM) of M1557 peptide were included in the medium. For the control purpose, blank reaction (GM medium only) and positive reaction (cell with GM containing Dexamethasone, a classic apoptosis inducer [16]) were also included. After 40 hours treatment, the cells were subjected to Caspase 3/7, 8, 9 activities measurement with Caspase-Glo assay kit (Promega, Madison USA). Briefly, the plates containing cells were removed from the incubator and allowed to equilibrate to room temperature for 30 minutes. 100 μl of Caspase-Glo reagent was added to each well, the content of well was gently mixed with a plate shaker at 300–500 rpm for 30 seconds. The plate was then incubated at room temperature for 2 hours. The luminescence of each sample was measured in a plate-reading illuminometer (Thermo Labsystems) with parameters of 1 minute lag time and 0.5 second/well read time. All Caspase activities were expressed as increase fold compared to untreated cells. The experiments were performed in triplicate and repeated on two separately-initiated cultures.

### Establishment of cellular proliferation assay for cancer cell lines

In order to establish a suitable cell proliferation assay for colon cancer cell line, human colon cancer HT-29 cells were seeded on 96 well plate with GM (McCoy's 5A medium containing 2 mM glutamine, 10% foetal bovine serum and 1% penicillin and streptomycin) at densities of 1250, 2500, 5000, 10000 and 20000 cells/well. Each cell density included 5 wells. Cells were incubated at 37°C in 5% CO_2_/95% air. Foetal bovine serum (FBS) has been proven to affect the result of Alamar blue assay [17] therefore after 48 hours of growth, GM was removed from each well and replaced with 200 μl of warmed fresh McCoy's 5A medium containing 2 mM glutamine and 1% penicillin and streptomycin (without FBS) plus 20 μl of Alamar Blue dye (Serotec, UK) which have been proven to be a new rapid and simple non-radioactive assay for cellular proliferation assessment [18]. Cells were further incubated at 37°C with 5% CO_2_/95% air for 5 hours. The fluorescence was measured with excitation at 530 and emission at 620 nm. The cellular proliferation in different cell density was then plotted.

### Validation of modified version of the Alamar Blue assay

To validated modified version of the Alamar Blue assay, a cell viability assay was carried out using CellTiter-Glo luminescent cell viability assay kit (Promega, Madison USA). Human colon cancer HT-29 cells were seeded on two parallel blank-walled 96-well plates with GM (McCoy's 5A medium containing 2 mM glutamine, 10% foetal bovine serum and 1% penicillin and streptomycin) at densities of 1250, 2500, 5000, 10000 and 20000 cells/well. The medium volume for each well was 100 μl. Cells were incubated at 37°C in 5% CO_2_/95% air for 48 hours; after incubation cells in one plate were directly subjected to the cell viability assay. Cells in another plate were subjected to the following procedures. Firstly GM was removed from each well and replaced with 100 μl of warmed fresh McCoy's 5A medium containing 2 mM glutamine and 1% penicillin and streptomycin (without FBS). Secondly cells were incubated at 37°C with 5% CO_2_/95% air for further 5 hours. After incubation the plate was subjected to the cell viability assay.

The procedures for cell viability assay for both plates were the same. Briefly, after incubation, plates were equilibrated to room temperature from 37°C for 30 minutes. 100 μl of CellTiter-Glo reagent (Promega, Madison USA) was added to each well and mixed for 2 minute on orbital shaker. The plate was incubated at room temperature for 10 minutes to stabilize luminescent signal. The luminescence of each sample was measured in a plate-reading illuminometer (Thermo Labsystems) with parameters of 1 minute lag time and 0.5 second/well read time. The experiments were performed in triplicate and repeated on two separately-initiated cultures.

### Effect of M1557 peptide on colon cancer cell growth

To evaluate the effect of M1557 peptide on colon cancer cell proliferation, human colon cancer HT-29 cells were seeded on a 96 well plate with McCoy's 5A medium containing 2 mM glutamine, 10% foetal bovine serum and 1% penicillin and streptomycin at density of 1 × 10^4 ^cells/well for 24 hours. The medium was then replaced with the same fresh medium plus different concentrations (0, 0.001, 0.01, 0.1 and 1 μM) of M1557 peptide. After 48 hours of exposure to the peptide, cells were then subjected to the modified Alamar Blue assay as described previously. Percentage inhibition of cell proliferation was determined using the following formula:

Growth inhibition (%) = Ft/Fc × 100

where Ft and Fc represent the units of fluorescence (RLU) in the treated and the control cells respectively.

In order to prove that the inhibition of M1557 on proliferation of HT-29 cells was due to the M1557 peptide blocking IGF-I receptors specifically, cell proliferation was also performed in serum free medium with addition of different concentrations of IGF-I. Briefly human colon cancer HT-29 cells were seeded onto 96 well plate with McCoy's 5A medium containing 2 mM glutamine, 10% foetal bovine serum and 1% penicillin and streptomycin at density of 1 × 10^4 ^cells/well for 24 hours. The experiment was performed in duplicate. For the first set of experiments the medium was replaced with McCoy's 5A medium containing 2 M glutamine and 1% penicillin-streptomycin and different concentrations (0, 1, 10 and 100 ng/ml) of recombinant human IGF-I (Peprotech EC Ltd, UK). For the second set, the medium was replaced with the same medium as the first set but with addition of 2 μM of M1557 peptide in each well. After 48 hours the cell proliferation was assessed with the method described as previously. For cell proliferation assay all samples were in triplicate and the experiments were repeated on two separately-initiated cell cultures.

### Protein extraction and Western Blotting

HT-29 cells were seeded in a 6 well plate either with GM (McCoy's 5A medium containing 2 mM glutamine, 10% foetal bovine serum and 1% penicillin and streptomycin) or GM containing M1557 peptide (1 μM and 2 μM) at density of 1 × 10^5 ^cells/well. After 1 week incubation, the supernatant was removed from each well. The attached cells were washed with PBS three times before protein extraction. Cellular proteins were extracted with the Ripa buffer containing protease inhibitor cocktail (Roche). Total protein concentration in the samples was determined using a modified Lowry protein assay kit (Pierce Biotechnology, USA). 20 μg of total protein (10μl in volume) were mixed with 10 μl of laemmi sample buffer containing 4% SDS, 20% glycerol, 10% 2-mercaptoethanol, 0.004% bromphenol blue and 0.125 M tris HCl, pH approx. 6.8 (Sigma, UK). The samples were then denatured by heating at 95°C for 5 minutes. Sodium dodecyl sulphate – polyacrylamide gel electrophoresis (SDS-PAGE) was used to separate the proteins, which were then electro-blotted on to polyvinylidene fluoride (PVDF) membrane (Bio-Rad, USA). The membrane was blocked with 5% milk in PBS (Marvel semi skimmed milk powder) and incubated with rabbit anti-human Bax polyclonal antibody (Santa-Cruz, USA) at 1/200 dilution followed by incubation with anti-rabbit IgG antibody conjugated with horse radish peroxidase (Dako, UK) at 1/2000 dilution. Each membrane was illuminated with Super Signal West Dura extended duration Substrate (Pierce, USA) and exposed to X-ray film for 10 seconds (Fuji, Japan). The X-ray films were scanned and the density of the protein bands were analysed with Bio-Rad densitometry software (Molecular Analyst, windows software for Bio-Rad image analysis system version 1.5, USA).

### Statistical Analysis

All data have been examined and followed a normal distribution. All results were expressed as mean ± SM. One way ANOVA (Prism version 4 2004 edition, USA) with multiple comparison test was used for statistical analysis and P < 0.05 is considered as significant and indicated as *; P < 0.01 is considered as higher significance and indicated as **. P > 0.05 is considered as not significant and marked as NS.

## Results

### M1557 peptide induced colon cancer cells apoptosis

**Figure 2:**
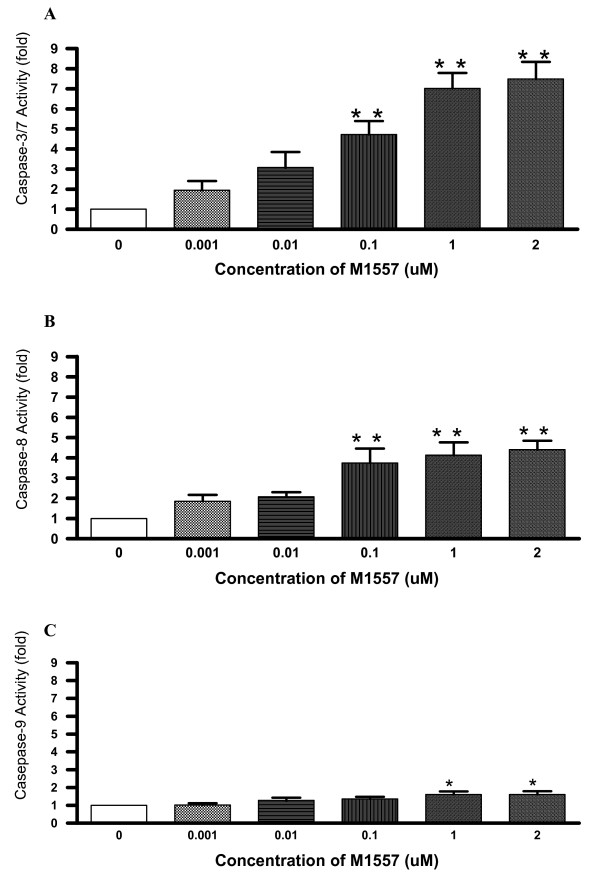
**Following treatment by M1557 peptide for 40 hours, human colon cancer cell (HT-29) caspase3/7 (A), 8(B) and 9 (C) activities increased in a dose response manner.** Caspase 3/7 activities in treated cancer cells increased 2–7 times compared to the untreated control cancer cells with significant effects at dose of 0.1 μM onward concentrations (P < 0.001). Caspase 8 activities in treated cancer cells also increased 2–5 times compared to the untreated control cancer cells, with significant effects at 0.1 μM onward concentrations (P < 0.001). Caspase 9 activities in treated cancer cells increased in a small scale (1.6 times), with significant effects at the higher concentrations (1 μM onward; P < 0.05) compared to caspase 3/7 and 8. Significance value: * P < 0.05; ** P < 0.001.

Following 40 hours incubation with M1557 peptide, caspase 3/7 (cysteine aspartic acid-specific protease) activities in colon cancer (HT-29) cells increased 2–7 fold over the dose range compared to the untreated control cancer cells. It is worthwhile to note that the M1557 peptide increased caspase 3/7 activities in a dose response manner with significant effects at 0.1 μM onward concentrations (P < 0.0001; Figure 2A). It has been recognized that caspase 3/7 activation is a central initiator and executioner of apoptosis in mammalian cells. Hence, M1557 treatment significantly increased cancer cell apoptosis.

It has been known that caspase 3/7 is activated by active caspase 8 or 9. We, therefore, also measured caspase 8, and 9 activities. The results showed that M1557 peptide increased caspase 8 activities 2–5 times over the dose range and the increase was also in a dose response manner with significant effects from 0.1 μM onward concentrations (P < 0.0001, Figure 2B). Caspase 8 activation is the step immediately following death-inducing signalling complex (DISC) formation. The latter is the part of extrinsic apoptosis pathway which involved death ligands bound to death receptors. The M1557 peptide also increased caspase 9 activities. This increase is again in a dose response manner with significant effects from 1 μM onward concentrations (P < 0.05, Figure 2C). However, it is interesting to note that M1557 peptide induced caspase 9 activities on a much smaller scale (1.2–1.6 times) compared to caspase 3/7 and 8. Caspase 9 activation is a part of intrinsic apoptosis pathways which involves cytochrome c release from the mitochondria [19] and is called mitochondria-mediated apoptosis. M1557 peptide increased caspase 3/7 and 8 activities by a large scale (2–7 fold for caspase 3/7, 2–5 fold for caspase 8). It appears that M1557 peptide induce cancer cells death mainly via the extrinsic apoptosis pathway. The intrinsic apoptosis pathway cannot be ruled out as M1557 peptide also increased caspase 9 activities by a smaller scale (1.2–1.6 folds) with higher concentrations.

### Establishment and validation of cellular proliferation assay for cancer cells

**Figure 3:**
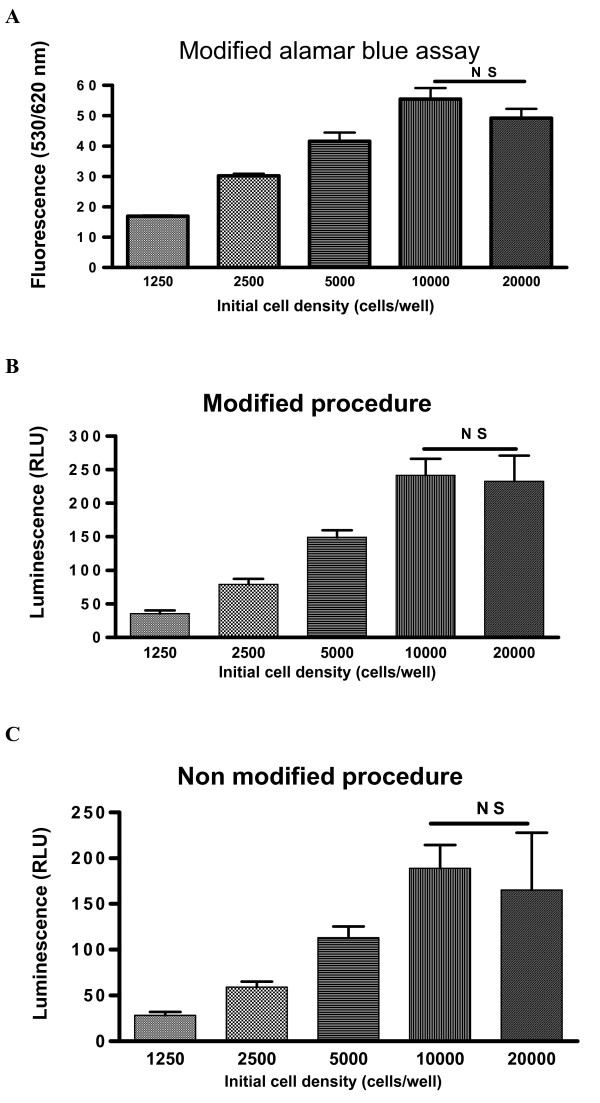
**HT-29 cellular proliferation was assessed at different cell densities (1250, 2500, 5000, 10000 and 20000 cells/well) with a modified Alamar Blue assay (A).** The increases of fluorescence at densities of 1250–10000 cells/well indicated the fluorescence readings designate the true cell number at these cell densities. The fluorescence at 20000 cells/well tended to decrease compared to that at 10000 cells/well, but the decrease was not statistically significant (P > 0.05, NS). To validate the modified version of Alamar Blue assay and to find whether the fluorescence drop at 20000 cells/well was a feature of the assay or cellular proliferation itself, two parallel procedures were performed simultaneously. One procedure (B) was the modified procedure similar to that in the modified Alamar Blue assay. Another procedure (C) was no modified procedure. The same range of cell densities (1250, 2500, 5000, 10000 and 20000 cells/well) were used in both procedures. At the end of two procedures, the cell numbers were assessed using CellTiter-Glo luminescent cell viability assay. Luminescence increased along with the increases of cell densities at 1250–10000 cells/well for both modified (B) and no modified (C) procedures. Luminescence at 20000 cell/well tended to decrease in both procedures, although the decreases were not statistically significant compared to that at 10000 cell/well density (P > 0.05; NS). Significance value: * P <0.05; ** P < 0.001; NS P > 0.05.

In order to assess cancer cell growth, a rapid and simple cellular proliferation assay is needed. Alamar Blue (Serotec, UK) has been proven to be a new rapid and simple non-radioactive dye for cellular proliferation assessment [18], but serum proteins and FBS used in cell culture were proven to interfere with Alamar Blue assay [17]. To avoid this interference, we have modified the assay by replacing GM medium (containing FBS) with the fresh warmed serum-free medium at the end of the treatment. Using this modified Alamar Blue assay to assess the different densities (1250, 2500, 5000, 10000 and 20000 cells/well) of HT-29 cancer cells, the results are shown in Figure 3A. In Figure 3A it is shown that the fluorescence (indicates of cell numbers) increased along with the increases of cell densities of 1250–10000 cells/well. This indicated that the fluorescence reading may define the true cell numbers in these cell densities. It should also be stated that the fluorescence decreased at 20000 cells/well density. The decrease was not statistically significant when comparing the reading at 20000 cell/well with that at 10000 cells/well (P > 0.05).

In order to validate the modified version of Alamar Blue assay and also to find whether the fluorescence drop at 20000 cells/well is a feature of the assay or cellular proliferation itself, cell viability assays were carried out. In cell viability assays, two parallel procedures were performed simultaneously. One procedure was the modified procedure similar to that in the modified Alamar Blue assay. Another procedure was a non modified procedure. At the end of two procedures, the cell numbers were assessed using the CellTiter-Glo luminescent cell viability assay. CellTiter-Glo luminescent cell viability assay is a homogeneous method of determining the number of viable cells in culture based on quantitation of the ATP present, which signals the presence of metabolically active cells. This method has been proven to be a accurate assay for cell proliferation and cytotoxicity [20]. The results of the parallel assays are shown in Figure 3B (modified procedure) and C (no modified procedure). In Figure 3B and 3C it can be seen that luminescence (indicator of the number of viable cells) increased along with the increases of cell densities between 1250–10000 cells/well for both procedures (modified and not modified). It is interesting to note that luminescence at 20000 cell/well decreased in both procedures, although the decreases were not statistically significant compared to that at 10000 cell/well density (P > 0.05). The similarity of results from both modified and not modified procedures indicated that the modified proliferation assay has not altered the proliferation of cells. The similar reading patterns among all three assays have also proven that the modified Alamar Blue assay is a reliable proliferation assessment for cancer cells. The fact that the readings seen at 20000 cells/well decreased in all three assays indicated that the drop in fluorescence or luminescence seen at 20000 cells/well was due to the cellular proliferation itself. The reasons for this drop may due to the facts that the high rate of cancer cell proliferation, plus the high starting density of cells will consume essential nutrients rapidly. A previous report similarly showed that nutritional deficiency caused human lung cancer cell arrest [21].

### M1557 peptide inhibits colon cancer cell proliferation

**Figure 4:**
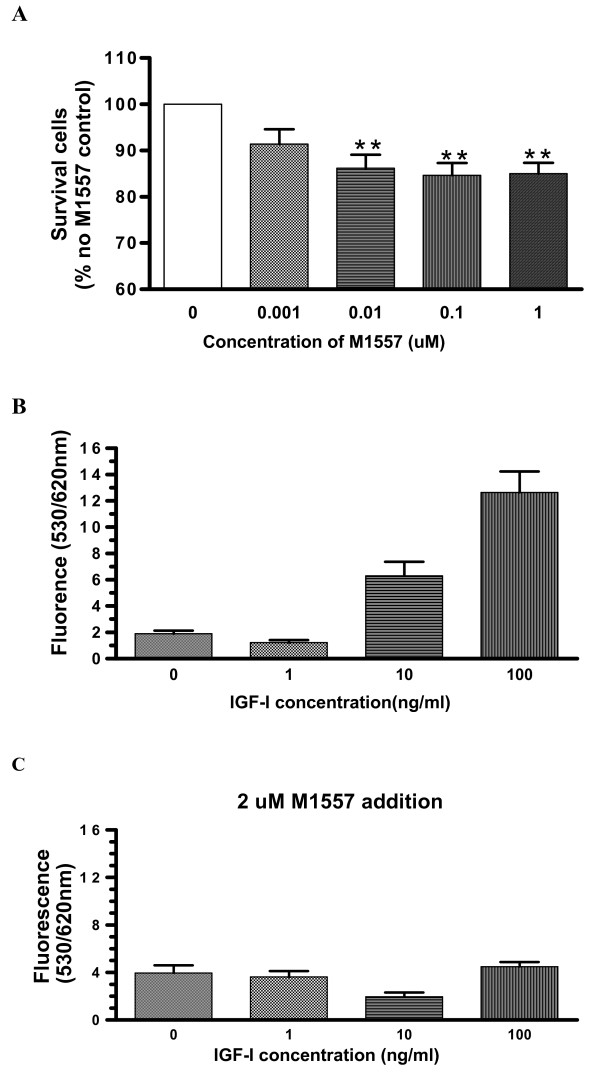
**After treating with M1557 peptide for 48 hours, cancer cell proliferation was inhibited by about 15% with significant effects at doses of 0.01 μM onward.** Again, this inhibition was in a dose responsive manner (A). To detect whether cellular proliferation inhibition was the result of interference with IGF-I signalling, two further experiments were performed with serum free medium. In the first set of experiments cells were treated with different concentrations of recombinant human IGF-I and it can be seen that IGF-I increased cancer cell proliferation in serum free medium. The increase was in a dose responsive manner (B). In the second set, cells were also treated the same as in the first set of experiments, but also with addition of 2 μM M1557 peptide in all samples. It can be seen that with M1577 inclusion, the IGF-I enhancing- proliferation dose response curve vanished (C). Significance value: * P < 0.05; ** P < 0.001.

Following 48 hour treatment with M1557 peptide, the proliferation of colon cancer cells was inhibited by 14–15% over the dose range of the peptide used in these studies. This inhibition showed a tendency towards a dose response relationship with significant effects from 0.01 μM onward concentrations (P < 0.01, Figure 4A).

In order to prove that the M1557 peptide inhibition was due to interference with IGF-I signalling, recombinant human IGF-I peptide was included in serum-free medium, which normally would yield an increase in tumour cell proliferation. The results showed that IGF-I indeed increased cancer cell proliferation in a dose response manner (Figure 4B). However when 2 μM of M1557 peptide was additionally included in all samples in a parallel experiment in which different dose of IGF-I peptide were used, the IGF-I enhanced proliferation response curves vanished (Figure 4C). All of these data indicated that M1557 peptide can inhibit HT-29 cell proliferation, and that this inhibition is likely to be due to its blockage of IGF-I signalling.

### M1557 increased the level of pro-apoptosis protein Bax

**Figure 5:**
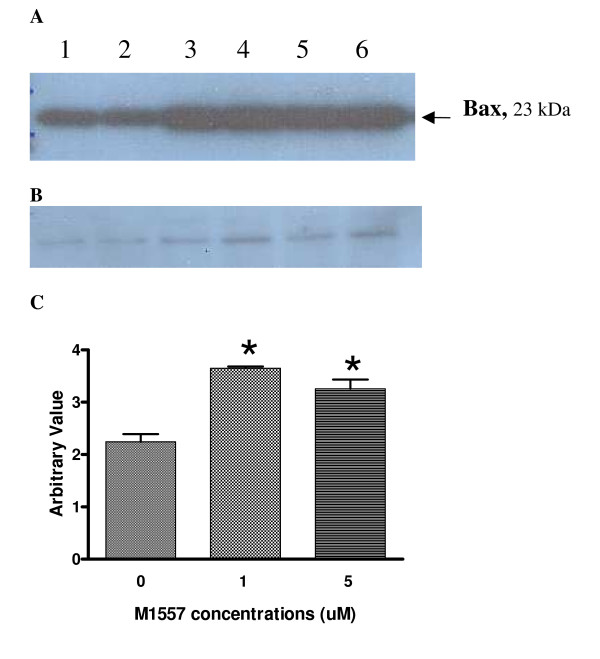
**Western blotting was used to detect pro-apoptosis Bcl2-associated X protein (Bax) in M1557 peptide treated and untreated cells (A).** After treatment with M1557 peptide at 2 μM and 5 μM concentrations, the level of Bax increased significantly compared to that in the untreated cells (P < 0.01) (C). (B) The level of β-actin in each sample was detected by a polyclonal anti-actin antibody (Sigma, UK). β-actin was used as internal standard to ensure the equal amounts of protein was loaded.

After 1 week's exposure to M1557 peptide, the level of Bax protein increased in M1557 treated cancer cells compared to untreated cells (P < 0.05 Figure 5A, C). Apoptosis is influenced by various intracellular proteins and enzymes. One important protein which influences apoptosis is Bcl2-associated X protein (Bax). Bax has been shown to promote apoptosis. The higher level of Bax in the M1557 peptide treated cancer cells indicates that M1557 increased apoptosis via Bax expression. Figure 5B showed the level of β-actin in each sample which was detected by a polyclonal anti-actin antibody (Sigma, UK). β-actin was used as internal standard to ensure the equal amounts of protein was loaded.

## Discussion

More than a decade ago it was hypothesized that cells with accelerated rate of proliferation are more liable to lead to the development of cancer [22]. A number of epidemiologic studies have shown that high levels of IGF-I are associated with increased risk for several common cancers, including those of breast [23], prostate [24], lung [25] and colorectal cancers [26]. Functionally, IGF-I not only stimulates cell proliferation but also inhibits cell apoptosis. Based on the IGF-I structure, we designed and synthesised a novel IGF-I receptor antagonist, M1557 peptide. We tested the M1557 peptide on human colon cancer (HT-29) cell apoptosis and proliferation. The results showed that this IGF-I D domain analogue increased cancer cell apoptosis, and in parallel inhibited cell proliferation. This action appeared to be a result of interfering with IGF-I signalling.

There are several potential interventions in the IGF- I signalling pathway such as application of inhibitory antibodies [10,27], anti-sense RNA constructs [28,29] and IGF-I analogue peptides [30,31]. IGF-I D domain has been proven to be an active site for IGF-I binding to its receptors [13,14], we therefore chose to synthesis a D domain analogue peptide. Although previous studies [30,31] have targeted the IGF-I C terminal, subsequent research using the same peptide [32,33] has provided uncertain information. In those reports two different peptides (one peptide was a partial analogue of IGF-I C terminal, while another has reversed sequences compared to the first one, but both peptides were named as JB3) were found to inhibit early renal tissue growth in diabetic and uninephrectomized rats but did not inhibit renal fibrosis, in which IGF-I is also involved [32,33].

The accumulated data from multiple systems have shown that IGF-I exerts a strong anti-apoptosis action on various cancer cells. Few studies have defined the putative mechanism by which the apoptosis process may be inhibited by IGF-I [34]. Apoptosis may proceed in two pathways: the intrinsic pathway and the extrinsic pathway. In the intrinsic pathway, stimulators such as stress, DNA damage or chemotherapy cause release of cytochrome c from mitochondria which consequently activate caspase-9 and caspase-3. The latter then triggers the apoptosis. The extrinsic pathway is caused by the direct interaction between so called "death ligands" and "death receptors", in which caspase-8 is activated. In M1557 peptide-treated cells, the caspase-8 activities were 2–5 times higher; caspase 3/7 were 2–7 times higher than untreated cells. In contrast, the caspase-9 activities increased by a relatively small scale (1.6 times) in the treated cells compared to the untreated cells. This suggested that the extrinsic apoptosis pathway may play the major role in the M1557 peptide-induced apoptosis in colon cancer cells, but the role of intrinsic apoptosis pathway cannot be completely ruled out. Further studies are needed to address this point.

It has been recognized that the combination of mitogenic and anti-apoptotic effects has a profound impact on tumour growth [35]. In our experiments, we found M1557 peptide treatment increased cancer cell apoptosis and inhibited cancer cell proliferation. Previous studies [30,31,36] also found that serum free-medium induced normal and malignant cells to undergo apoptosis and that addition IGF-I prevented the actuation of apoptosis. These findings suggested that blockade of the IGF-I receptor inhibited cell proliferation, perhaps as a result of the combination of anti-mitogenic and pro- apoptotic effects.

## Conclusion

Treating human colon cancer cells with a novel IGF-I D domain analogue (M1557 peptide) increased cancer cell apoptosis and inhibited cancer cell proliferation. Further examination of different caspase activities suggests that the extrinsic apoptosis pathways may play the major role in M1557 peptide-induced apoptosis in colon cancer cells. These results suggest that specific IGF-I receptor antagonists may have the potential to be developed as a novel therapy for colon cancers in the future.

## Abbreviations

IGF-I – insulin-like growth factor I; DISC- death-inducing signalling complex, FBS – foetal bovine serum; PBS – phosphate buffered saline; HPLC – high-pressure liquid chromatography; ACM – Acetamidomethyl; SDS-PAGE – sodium dodecyl sulfate polyacrylamide gel; RIPA – radio immuno precipitation assay; PVDF – polyvinylidene fluoride

## Competing interests

The author(s) declare that they have no competing interests.

## Authors' contributions

SYY and MCW conceived the study. SYY designed the experiments, carried out the study and prepared the manuscript. KMS**, **BF**, **AMS and MCW participated in the design, reviewed all data, and contribute in the preparation of the manuscript. All authors read and approved the final manuscript.
